# Distinct subdivisions of the cingulum bundle revealed by diffusion MRI fibre tracking: Implications for neuropsychological investigations

**DOI:** 10.1016/j.neuropsychologia.2012.11.018

**Published:** 2013-01

**Authors:** D.K. Jones, K.F. Christiansen, R.J. Chapman, J.P. Aggleton

**Affiliations:** a CUBRIC, School of Psychology, Cardiff University, Cardiff, CF10 3AT, United Kingdom; b Cardiff Neuroscience and Mental Health Research Institute, Cardiff University, Cardiff, CF10 3AT, United Kingdom

**Keywords:** Cingulate cortex, Hippocampus, Prefrontal cortex, Retrosplenial cortex, White matter

## Abstract

The cingulum is a prominent white matter tract that supports prefrontal, parietal, and temporal lobe interactions. Despite being composed of both short and long association fibres, many MRI-based reconstructions (tractography) of the cingulum depict an essentially uniform tract that almost encircles the corpus callosum. The present study tested the validity of dividing this tract into subdivisions corresponding to the ‘parahippocampal’, ‘retrosplenial’, and ‘subgenual’ portions of the cingulum. These three cingulum subdivisions occupied different medial–lateral locations, producing a topographic arrangement of cingulum fibres. Other comparisons based on these different reconstructions indicate that only a small proportion of the total white matter in the cingulum traverses the length of the tract. In addition, both the radial diffusivity and fractional anisotropy of the subgenual subdivision differed from that of the retrosplenial subdivision which, in turn, differed from that of the parahippocampal subdivision. The extent to which the radial diffusivity scores and the fractional anisotropy scores correlated between the various cingulum subdivisions proved variable, illustrating how one subdivision may not act as a proxy for other cingulum subdivisions. Attempts to relate the status of the cingulum, as measured by MRI-based fibre tracking, with cognitive or affective measures will, therefore, depend greatly on how and where the cingulum is reconstructed. The present study provides a new framework for subdividing the cingulum, based both on its known connectivity and MRI-based properties.

## Highlights

► The cingulum bundle was subdivided using DTI tractography. ► Distinct subgenual, retrosplenial, and parahippocampal subregions were differentiated. ► These subregions differed in their fractional anisotropy and radial diffusivity. ► Only a small proportion of fibres traverse the length of the human cingulum bundle. ► For neuropsychological studies the cingulum should not be seen as a unified tract.

## 1 Introduction

The cingulum bundle is a prominent white matter tract that extends longitudinally above the corpus callosum. At its rostral limit the cingulum curves around the front of the genu of the corpus callosum while caudally it curves behind the splenium. Studies into the functional importance of the fibres in this bundle have been aided considerably in recent years by the relative ease with which the cingulum is revealed by diffusion MRI-based fibre tracking, i.e., tractography (Catani, Howard, Pajevic, & Jones, 2002; Jones, 2008). Such studies have examined the status of the cingulum bundle in conditions such as depression, traumatic brain injury, Mild Cognitive Impairment, Alzheimer’s disease, and schizophrenia (e.g., Cullen et al., 2012; Jones et al., 2005a, 2006; Keedwell, Chapman, Christiansen, & Jones, 2012; Kubicki et al., 2003; Wu et al., 2010; Zhang et al., 2007).

Descriptions of the cingulum bundle have a long history, and it has been appreciated for over a century that the bundle contains many short association fibres, as well as longer fibres that potentially link the frontal lobe with the temporal lobes (Beevor, 1891; Brodal, 1981; Schmahmann & Pandya, 2006). Detailed information about the composition of the primate cingulum bundle arrived with the introduction of axonal tracer studies in monkeys (Baleydier & Mauguiere, 1980; Goldman-Rakic, Selemon, & Schwartz, 1984; Morris, Petrides, & Pandya, 1999a; Morris, Pandya, & Petrides, 1999b; Mufson & Pandya, 1984; Vogt & Pandya, 1987; Vogt, Pandya, & Rosene, 1987). Such studies confirmed that the cingulum contains many afferent and efferent fibres associated with the rostral, mid, and caudal cingulate cortices (e.g., areas 23, 24, 25, 29, 30, 31, 32). These fibres include connections with sites such as the anterior thalamic nuclei, lateral dorsal thalamic nucleus, dorsolateral prefrontal cortex, and insula (Domesick, 1970; Goldman-Rakic et al., 1984; Mufson & Pandya, 1984; Petrides & Pandya, 2006; Vogt & Pandya, 1987). Other cingulum fibres are connected to structures in the temporal lobe, including the parahippocampal cortices, subicular cortices, and amygdala (Goldman-Rakic et al., 1984; Morris et al., 1999b; Mufson & Pandya, 1984). As a consequence, the cingulum bundle forms a complex tract comprised of many different connections with trajectories of different lengths (Schmahmann & Pandya, 2006). Due to its many short fibres, it is likely that different parts of the cingulum are principally composed of distinct white matter populations that are likely to reflect different underlying functions.

The complex composition of the cingulum is, however, rarely reflected in published diffusion MRI-based tractography images of the tract. These images often show a continuous band of white matter that seemingly links, uninterrupted, the medial temporal lobe with retrosplenial, anterior cingulate, prefrontal, and subgenual areas (e.g., Catani et al., 2002; Concha, Gross, & Beaulieu, 2005; Gong et al., 2005; Singh & Wong, 2010; Thiebaut de Schotten, Dell’Acqua, Valabregue, & Catani, 2012; Xie et al., 2005). Such images suggest an apparent continuity of fibres in the cingulum bundle, while anatomical tracing studies in nonhuman primates reveal the presence of numerous short association fibres (e.g., Mufson & Pandya, 1984).

This discrepancy may arise as an artifact of the way that tractography data are compiled. A common approach is to reconstruct multiple virtual fibre pathways (perhaps from every voxel in the dataset), and then to use anatomical regions of interest (ROIs) as ‘waypoints’ to ‘virtually dissect’ out the tract of interest (Conturo et al., 1999; Catani et al., 2002). Such ROIs can be used inclusively (e.g., the tract has to pass through multiple regions of interest to be retained for analysis) or exclusively (e.g., if the tract passes through this region, then it should be rejected). In keeping with Boolean logic, the inclusive ROIs are named ‘AND’ gates, and the exclusive ROIs as ‘NOT’ gates.

The most common practice of visualizing the cingulum bundle with tractography is to put single or multiple regions of interest dorsal to the body of the corpus callosum and to identify and retain those pathways that pass through the ROIs (Catani et al., 2002; Concha et al., 2005; Gong et al., 2005; Singh & Wong, 2010; Thiebaut de Schotten et al., 2012; Xie et al., 2005). A concern is that the cingulum bundle may actually comprise several, largely distinct subdivisions that only appear united due to the numerous short association fibres within this tract and the resulting overlap in their trajectories. The present study selected three potential subdivisions within the extent of the cingulum bundle (‘parahippocampal’, ‘retrosplenial’, and ‘subgenual’). One of these subdivisions, the parahippocampal subdivision, was visualized in two different ways. One parahippocampal reconstruction (‘unrestricted’) used very similar logic to that applied to the other two potential cingulum subdivisions (subgenual and retrosplenial), and was intended to reveal the full extent of the tract. The second parahippocampal reconstruction (‘restricted’) was intended to segregate any parietal and occipital fibres, and so a ‘NOT’ gate was used to remove more rostral connections, e.g., those with the frontal lobe. For this reason, the second reconstruction is designated as the ‘restricted’ parahippocampal subdivision. The goal was to subdivide the cingulum even further to help isolate potential subdivisions at a finer level.

The questions addressed by this study included whether MRI-based tractography could help determine if these three cingulum subdivisions are likely to contain different fibre populations, and whether there are topographical differences within the tract. A further goal was to compare other characteristics, e.g., fractional anisotropy or radial diffusivity, across these same subdivisions. One purpose was to determine whether neuropsychological investigations that relate cingulum bundle status with cognition should focus on specific tract subdivisions or whether it is acceptable to generalize along the extent of the tract.

## 2 Materials and methods

### 2.1 Participant recruitment

Twenty right-handed women (mean age at scan=36.3 years, range 27–42) were recruited from the Cardiff Community panel, a cohort of volunteers drawn from the wider community that had agreed to be contacted about studies in the University. To avoid ongoing maturation effects, we limited our age range to >25 years, and to avoid documented ageing effects on diffusion MRI metrics, set an upper limit of 45 years. Finally, to reduce possible sources of variance, we opted to recruit a single gender. In this case, 20 right-handed females that satisfied the criteria were available from the panel. All participated under informed consent and the study was approved by the Ethics Committee of the School of Psychology in Cardiff University. Usual contraindications for MRI were applied (e.g., metallic implants, pacemakers, claustrophobia), and all participants were free from known neurological or psychiatric conditions.

### 2.2 Diffusion MRI scanning

Diffusion weighted MR data were acquired on a 3 T GE HD_*x*_ MRI system (General Electric Healthcare) with a peripherally-gated twice-refocused spin-echo echo-planar imaging sequence providing whole oblique axial (parallel to the commissural plane) brain coverage. Data were acquired from 60 slices of 2.4 mm thickness, with a field of view of 23 cm, and an acquisition matrix of 96×96 (yielding isotropic voxels of 2.4×2.4×2.4 mm, reconstructed to a resolution of 1.9×1.9×2.4 mm). TE (echo delay time) was 87 ms and parallel imaging (ASSET factor=2) was employed. Diffusion encoding gradients (*b*=1200 s/mm^2^) were applied along 60 isotropically-distributed directions (Jones, Horsfield, & Simmon, 1999) and six additional non-diffusion weighted scans were collected. The acquisition time was approximately 26 min.

### 2.3 Diffusion MRI data pre-processing

The data were corrected for distortions and subject motion using an affine registration to the non-diffusion-weighted images, with appropriate re-orienting of the encoding vectors (Leemans & Jones, 2009). A single diffusion tensor model was fitted (Basser, Mattiello, & LeBihan, 1994) to the data to allow quantitative parameters such as fractional anisotropy (FA) and radial diffusivity to be computed. Maps of FA were constructed for each participant. Constrained spherical harmonic deconvolution (CSD) was used to estimate the fibre orientation density function (fODF) in each voxel (Tournier, Calamante, Gadian, & Connelly, 2004).

### 2.4 Tract reconstructions

**Fig. 1:**
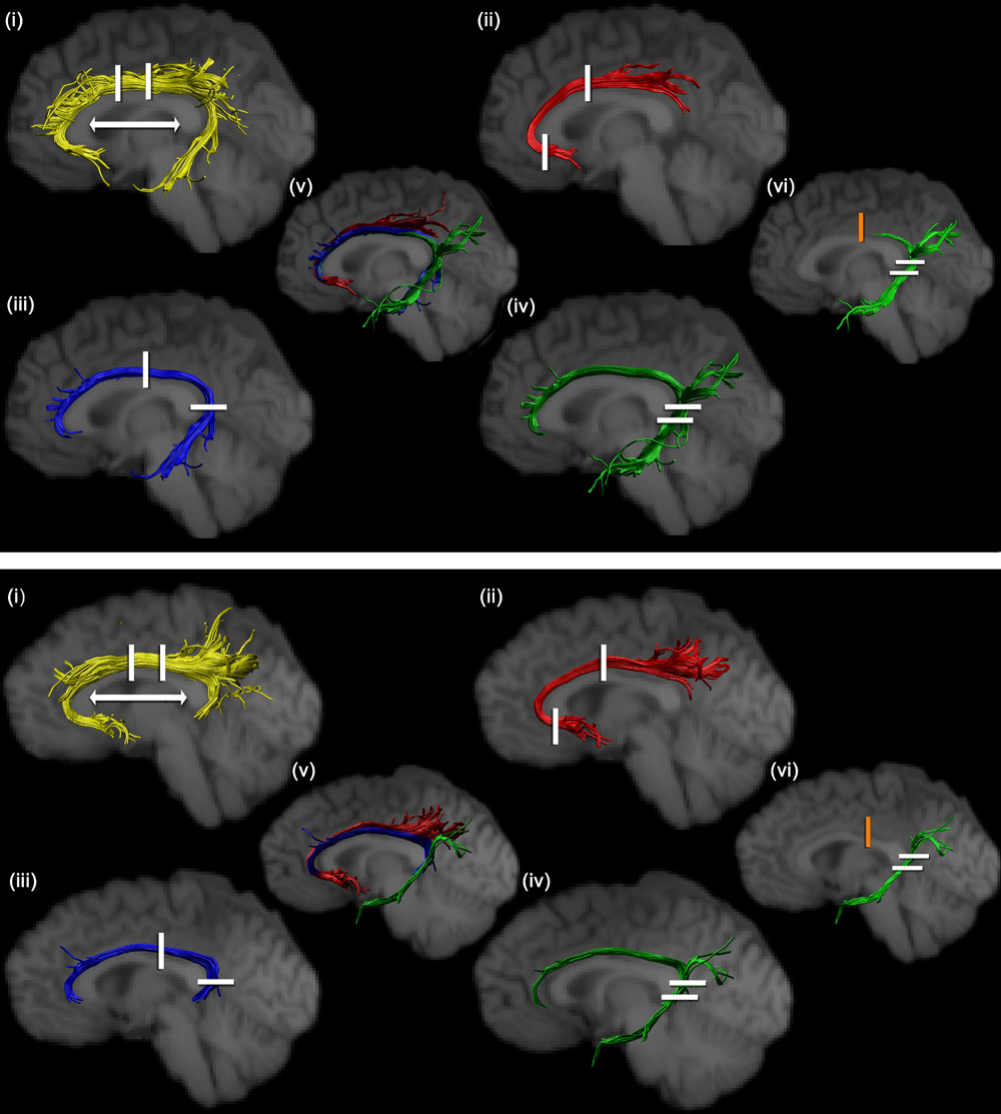
Cingulum reconstructions for two individual participants (upper and lower). The data shown in Fig. 1 upper are from the left hemisphere, the data in Fig. 1 lower are from the right hemisphere. The various parasagittal views show: (i) the ‘standard cingulum’, (ii) the ‘subgenual cingulum’, (iii) the ‘retrosplenial cingulum’, (iv) the ‘unrestricted parahippocampal cingulum’, and (vi) the ‘restricted parahippocampal cingulum’. The hemisphere placed in the centre (v) shows the relative positions of the subgenual, retrosplenial, and parahippocampal cingulum regions when they are overlaid in each individual case. The arrow in section (i) points to the two sites used to determine the mid rostral–caudal point of the corpus callosum. It can be seen from sections (ii)–(vi) that these tracts do not appear to occupy the same space around the corpus callosum. The locations of AND ROIs are shown as white bars, while the one NOT ROI (restricted parahippocampal cingulum, (vi) is shown as an orange bar.

Deterministic tractography was carried out using *ExploreDTI* (Leemans, Jeurissen, Siibers, & Jones, 2009) following peaks in the fODF reconstructed from CSD (Jeurissen, Leemans, Jones, Tournier, & Sijbers, 2011). For each voxel in the data set, streamlines were initiated along any peak in the fODF that exceeded an amplitude of 0.1 (thus, multiple fibre pathways could be generated from any voxel). Each streamline continued, in 0.5 mm steps, following the peak in the ODF that subtended the smallest angle to the incoming trajectory. The termination criteria included: a turning angle of greater than 60° and an fODF amplitude threshold of 0.1. Once the ‘whole brain tractography’ was complete, regions of interest were drawn on the map of fractional anisotropy of each participant and subsequently used to dissect the cingulum bundle according to five closely-related protocols (Fig. 1).

All tract reconstructions were performed independently by two experimenters (KC, RC). For each reconstruction, the mean fractional anisotropy (FA) and mean radial diffusivity (RD) were obtained by averaging the FA and RD values sampled at 0.5 mm steps along the entire length of the tract (Jones, Travis, Eden, Pierpaoli, & Basser, 2005b). Prior to any systematic data collection, the two experimenters ran an initial set of pilot reconstructions using variable temporal lobe ‘AND’ gates. For the final reconstructions, specification of the locations for the AND and NOT gates was fixed against particular landmarks, so aiding the reproducibility of tract reconstruction.

For all of the various subdivision reconstructions the corpus callosum was first identified on the midsagittal slice. The next step was to find the parasagittal level in each hemisphere that provided the most extensive visualisation of the cingulum bundle. The position of the corpus callosum in that same plane was then used to derive a set of fixed landmarks for subsequent ROIs. The first reconstruction (‘standard cingulum’) adopted the inclusive strategy used in many studies whereby much of the full extent of the cingulum is visualized.

#### 2.4.1 ‘Standard cingulum’ reconstruction (Fig. 1i)

The rostral–caudal midpoint of the body of the corpus callosum was first identified (Fig. 1i). This point was defined as the mid-way point between the back of the curve of the genu (i.e., its most posterior part at the flexure) and the front of the splenium (i.e., its most anterior part at the flexure). These callosal sites are indicated by the arrows in Fig. 1i. From this midpoint, the coronal sections that were five slices anterior and five slices posterior were identified (Fig. 1i). These two sections were, therefore, separated by approximately 18 mm in the rostral–caudal plane. All streamlines that passed through both regions of interest were retained as ‘cingulum’ pathways (Fig. 1i, see also Catani et al., 2002; Gong et al., 2005; Singh & Wong, 2010; Xie et al., 2005).

This procedure was repeated in each hemisphere for all 20 participants. As will be discussed later, a probabilistic overlay of the tract reconstructions from all 20 participants was made without the use of the further regions of interest. However, for the illustrations in Fig. 1, additional ‘NOT’ ROIs were used to exclude tracts that were inconsistent with known projections of the cingulum.

#### 2.4.2 ‘Subgenual’ subdivision reconstruction (Fig. 1ii)

Two AND ROIs were employed. One ROI was in exactly the same location as the rostral ROI used for the standard cingulum reconstruction (Fig. 1i). The second ROI was placed in the subgenual part of the cingulum (Fig. 1ii). This ROI was placed on the third coronal slice caudal to the most anterior part of the genu, i.e., its anterior limit. “NOT” ROIs were occasionally placed after visual inspection to exclude any outlier tracts that were inconsistent with the known anatomy of the cingulum bundle. All other aspects of tract reconstruction matched those for the standard cingulum.

#### 2.4.3 ‘Retrosplenial’ subdivision reconstruction (Fig. 1iii)

Two AND ROIs were used for dissecting the ‘retrosplenial’ subdivision. One ROI was placed in the same location as the more caudal ROI used for the standard cingulum (Fig. 1i). The location of the second ROI was determined by finding the most ventral plane of the splenium and identifying the horizontal section that was three or four slices (∼6 mm) above the base of the splenium (Fig. 1iii). All other aspects of tract reconstruction matched those for the subgenual division.

#### 2.4.4 ‘Parahippocampal’ subdivision reconstructions (‘unrestricted’ and ‘restricted’) (Fig. 1iv and vi)

Two AND ROIs were placed behind and below the splenium. The upper of these two ROIs was in the same location as the caudal ROI used for the retrosplenial cingulum (Fig. 1iii). The second AND ROI was placed four horizontal slides below this upper AND gate (Fig. 1iv). The parahippocampal subdivision of the cingulum was then reconstructed in two different ways (‘unrestricted’ and ‘restricted’). For the ‘unrestricted’ reconstruction there were just two AND gates in the temporal lobe (Fig. 1iv). For the ‘restricted’ reconstruction an additional NOT gate was placed above the body of the corpus callosum (Fig. 1vi) in the same position as the more caudal of the two AND gates used for the standard cingulum reconstruction (Fig. 1i). The purpose of the NOT gate was to help isolate those pathways caudal to the splenium and so help to determine whether they have distinct properties. The results of the comparisons using these two sets of parahippocampal cingulum reconstructions are separated within the Results section. The initial series of statistical comparisons among the three different cingulum subdivisions focused on the unrestricted parahippocampal reconstruction as this version, like those for the other subdivisions, only used AND gates and so should have the most overlap with the other reconstructions. In contrast, the ‘restricted’ parahippocampal subdivision reconstruction should favour those fibres in this tract that are interconnected with parietal and occipital regions, so more fully testing the extent to which this tract may be heterogeneous.

### 2.5 Tract overlap maps to assess inter-subject agreement

Each tract reconstruction was ‘binarized’ by simply scoring a voxel in a matrix (of the same size as the FA map) as one or zero—according to whether a streamline intersected it (1) or not (0).

To ascertain the level of spatial overlap between the tract reconstructions made by the two experimenters (KC, RC), the Dice coefficient (Dice, 1945) was computed between each pair of binarized tract maps for each participant. (Note that a score of one was assigned to a voxel regardless of whether just one streamline, or 100 streamlines intersected the voxel, which is to borne in mind when interpreting the Dice coefficients).

The FA map was transformed to Montreal Neurological Institute (MNI) space, using the FMRIB58_FA template provided as part of the FSL software package (www.fmrib.ox.ac.uk/fsl). All subjects’ FA data were then aligned into a common space using the nonlinear registration tool FNIRT (Andersson, Jenkinson and Smith (2007a, 2007b)), which uses a *b*-spline representation of the registration warp field (Rueckert et al., 1999). The transformations from the registration procedure were then applied to the binarized tract maps to take them into MNI space.

For each tract reconstruction, in each hemisphere, the 20 normalized tract maps were then averaged (arithmetic mean)—and thresholded at multiple thresholds from 10 to 100%, in steps of 5%, (where the threshold for each voxel represents the proportion of participants who had at least one streamline intersecting that voxel). The Dice coefficient (Dice, 1945) was again used to quantify the level of agreement between the two ‘probabilistic’ maps at these different thresholds.

### 2.6 Statistical analyses

The first set of analyses concerned the inter-observer reliability of the two experimenters (intra-class correlations and *t-*tests). The alpha level for these multiple intra-class correlations was kept at *p*<0.05 as it is the consistency across these correlations that is most informative, and not the significance of individual correlations.

A series of within-group *t*-tests then compared the fractional anisotropy measures (*n*=20) derived by the two experimenters for the six tract regions (three areas, left and right hemisphere). Here, the question was whether any sets of scores differed (suggesting a systematic difference between observers). For this reason, alpha remained at *p*<0.05 as this level provides a more stringent test of whether the scores differed between the two observers, i.e., reducing alpha would have made it more likely that the observers’ scores would appear to agree.

Within group comparisons (matched sample *t*-tests, two tailed) then compared the fractional anisotropy and radial diffusivity measures in the three white matter regions of interest. For each measure (fractional anisotropy or radial diffusivity) there were a total of nine comparisons, comprising three comparisons across hemispheres for the same subdivision (e.g., left subgenual subdivision versus right subgenual subdivision) and six comparisons across subdivisions in the same hemisphere (three for each hemisphere). The alpha level for these comparisons was adjusted accordingly with the Bonferroni method (Howell, 1995) to give a significance level of *p*≤0.0056. Likewise, correlations were examined between the mean fractional anisotropy (and the radial diffusivity) scores of each tract subdivision in the same hemisphere and with the same tract subdivision across hemispheres, making a total of three comparisons for each subdivision (nine in total). For this reason, alpha was set at *p*≤0.0056.

### 2.7 Creation of generic ROIs in standard space

As a move towards fully-automated virtual dissection of the various cingulate subdivisions, and to facilitate further studies by others, we sought to generate ‘standardized’ regions of interest in standard (MNI) space, such that when transformed to each participant’s native space, and applied to their whole brain tracking result, the same three sections could be isolated. To generate these generic ROIs, the following procedure was adopted:

(a) For each participant, and for each ROI, the voxels contained within the ROI were first identified. (b) The participant’s FA map was warped nonlinearly to the FMRIB58_FA template in the FSL software suite. (c) The inverse of this transformation was then computed (using the *invwarp* tool from the FSL software suite). (d) The forward transformation was then applied to the ROI voxels to take them into MNI space. (e) Finally, each warped-ROI was then ‘binarized’ (1’s inside the ROI, zeros outside). (f) Steps (a)–(e) were then repeated for each participant, and the transformed ROIs overlaid on top of each other. The resulting overlay map was used to create generic ROIs that encompassed every non-zero voxel from the individual (warped) ROIs from each participant.

Steps (a)–(f) were repeated for each ROI shown in Fig. 1 to generate a set of ROIs. To test the effectiveness of the ‘standard space ROIs’ thus created, the inverse warps computed at step c in the procedure for each participant, were applied to warp the standardized ROI to native space. These new ROIs were then used to constrain the whole brain fibre tracking result, and compared visually with the results obtained when ROIs were drawn in the native space of the participant.

## 3 Results

### 3.1 Inter-observer reliability

All tracts were successfully reconstructed by both observers (KC, RC), allowing for an assessment of inter-observer reliability. Three potentially separate subdivisions were visualized in each hemisphere (subgenual, retrosplenial, parahippocampal), i.e., six in total.

Table 1 in Appendix A shows the average (of 20) Dice coefficients computed for each of the subdivisions in the two hemispheres, where it can be seen that the mean Dice score never drops below 0.84.

For the analyses of microstructural parameters (Section 3.1) the ‘restricted’ rather than the ‘unrestricted parahippocampal subdivision measurements are typically reported as this version has more steps, and so could potentially generate more variability. The first analyses compared the mean fractional anisotropy (FA) for each of these six areas of white matter, from each of the twenty participants measured by the two observers. High intra-class correlations were found for FA, which ranged from 0.79 (left subgenual subdivision) to 0.92 (right subgenual subdivision). (Others were: 0.83 left retrosplenial subdivision; 0.84, right restricted parahippocampal subdivision; 0.91, right retrosplenial subdivision; 0.91, left restricted parahippocampal subdivision). As a consequence, all six correlations were significant (all *p*<0.01).

It was also possible to compare directly the six sets of mean fractional anisotropy scores derived by each observer. Paired *t*-tests (df 19) revealed no significant differences between the mean fractional anisotropy scores derived by the two observers (all *p*>0.1 except for right subgenual subdivision, where *p*=0.079). The high intra-class correlations help to confirm that the methods for quantifying different subdivisions of the cingulum were highly reliable, while the *t*-tests show that the absolute FA scores for the two observers were comparable.

### 3.2 Tract reconstructions

Fig. 1 illustrates reconstructed sets of tracts for two individual participants. The figure not only shows the different locations and extent of the various subdivision reconstructions (including the ‘standard reconstruction’ and the ‘restricted parahippocampal subdivision’), but also shows some of the individual variability between two of the 20 participants.

Anatomical differences between all three cingulum subdivisions were visible in both hemispheres in all twenty participants. In addition, individual differences were often evident as, for example, in the way that the reconstructed retrosplenial subdivision extended by differing degrees into the temporal lobe (Fig. 1iii upper versus lower). For these reasons, the main findings are derived from the overlap (co-registration) maps of the three areas of white matter in each hemisphere (subgenual, retrosplenial, parahippocampal).

**Fig. 2:**
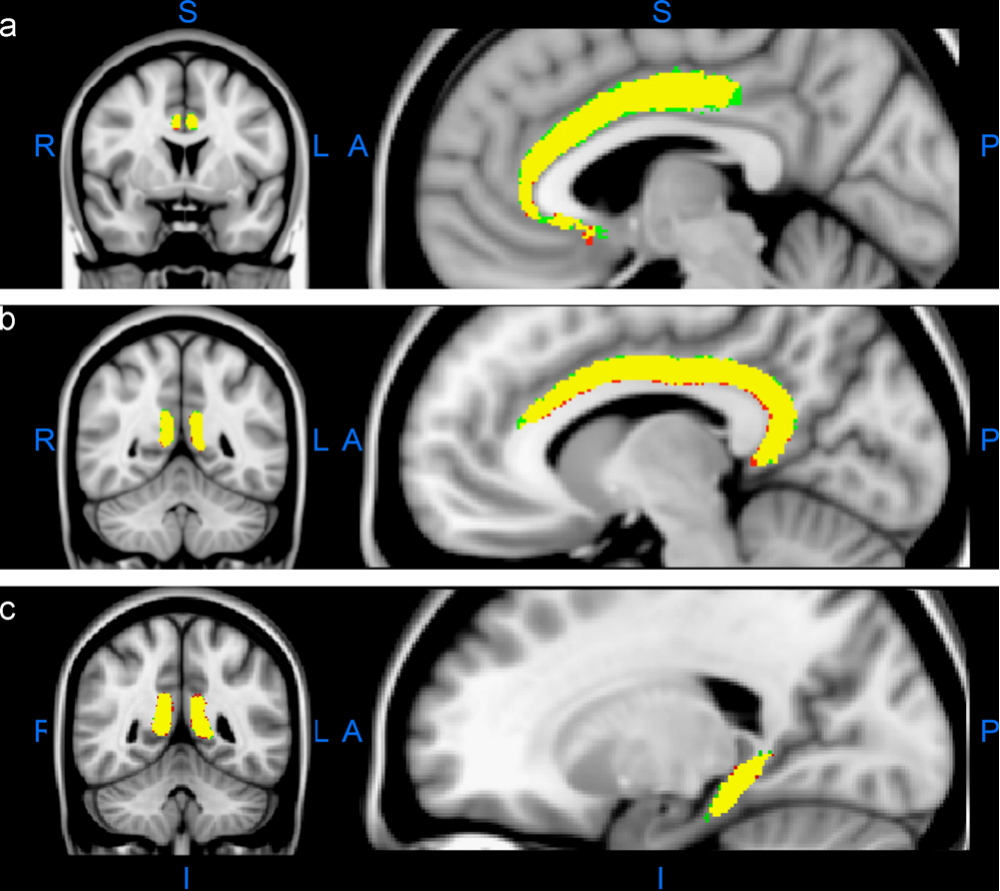
Probability maps of Observer 1 (KC, red) and Observer 2 (RC, green) and their combined output (yellow) for: (a) subgenual, (b) retrosplenial, and (c) parahippocampal (unrestricted) cingulum subdivisions. The areas in yellow are common to both observers, so that any inter-observer discrepancies are depicted in red and green. The sections in the left column are coronal, those in the right column are parasagittal. Abbreviations: A, anterior; I, inferior; L, left; P, posterior; R, right; S, superior. (For interpretation of the references to color in this figure legend, the reader is referred to the web version of this article.)

The co-registration procedure made it possible to overlay the location of a given tract derived from all 20 participants by one observer with the same tract derived by the other observer. Consequently, an additional way of assessing the reliability of the DTI procedures was to compare directly the spatially-normalized tracts derived by the two observers. The outcome of this overlay procedure for three subdivision reconstructions (subgenual, retrosplenial, unrestricted parahippocampal) is shown in Fig. 2. The population reconstruction by Observer 1 is in red and that of Observer 2 is in green. These reconstructions were overlaid to reveal those voxels (shown in yellow) where both observers had recorded white matter. It is immediately evident that there was very little variation between the final group subdivisions (subgenual, retrosplenial, unrestricted parahippocampal) derived by the two observers (Fig. 2, upper, mid, lower, respectively). The same overlay procedure was also used for the standard cingulum (Supplemental Fig. 1), where again there was an extremely high level of agreement.

**Fig. 3:**
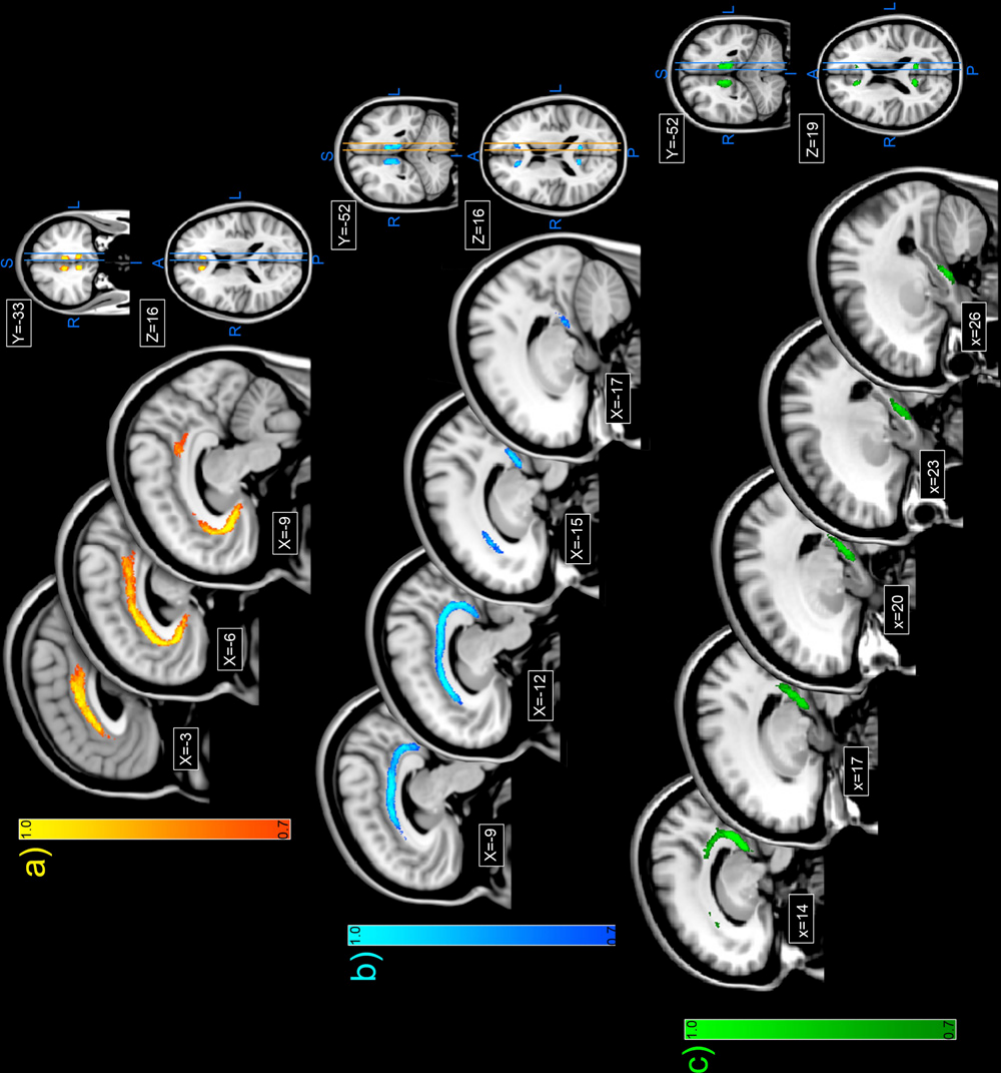
Topology of the individual subdivisions, together with MNI co-ordinates, highlighting the topographic organization of the sub-regions. The figure shows population reconstructions for the subgenual cingulum [(a) upper], retrosplenial cingulum [(b) mid], and parahippocampal (unrestricted) cingulum [(c) lower]. Each section shows in colour the region where 70% or more of the 20 cases had evidence of cingulum white matter within a given voxel. The extent of agreement above 70% is indicated by colour gradation such that those areas in yellow (subgenual), turquoise (retrosplenial), and light green (parahippocampal) had the highest level of agreement across cases. In contrast, those areas in red (subgenual), blue (retrosplenial), and dark green (parahippocampal) were closest to the 70% threshold. The location of the individual sections is given by their MNI coordinates. The pairs of parallel lines depict the positions of the most medial and most lateral parasagittal sections depicted for that cingulum subdivision. All data depicted are from one observer. Abbreviations: A, anterior; I, inferior; L, left; P, posterior; R, right; S, superior. (For interpretation of the references to color in this figure legend, the reader is referred to the web version of this article.)

**Fig. 4:**
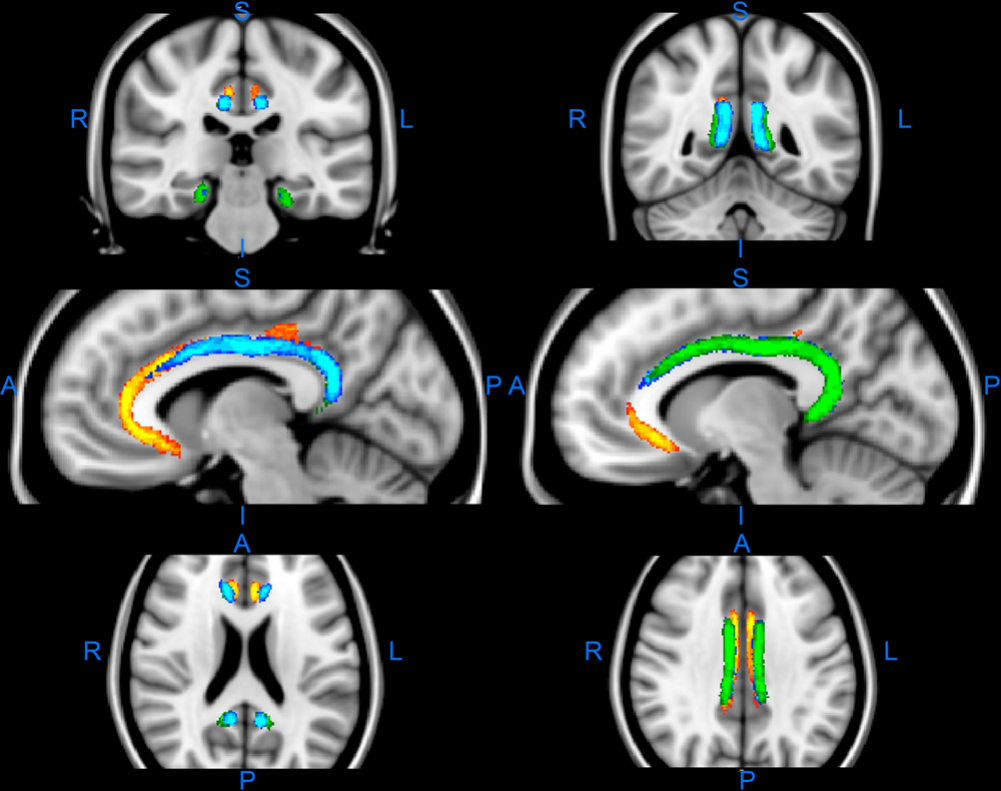
Composite showing the overlay of the subgenual (yellow), retrosplenial (blue), and unrestricted parahippocampal (green) cingulum reconstructions on top of each other. All conventions as for Fig. 3. The sections are in coronal (upper), parasagittal (mid), and horizontal (lower) planes. Abbreviations: A, anterior; I, inferior; L, left; P, posterior; R, right; S, superior. (For interpretation of the references to color in this figure legend, the reader is referred to the web version of this article.)

The next step was to compare the relative locations of these various cingulum subdivisions, in particular at those levels where they appear to occupy common space, e.g., above the body of the corpus callosum. These inter-subdivision comparisons used the data derived by one observer (the results are qualitatively identical for both observers). Figs. 3 and 4 show in three planes and at multiple levels the probabilistic estimates for the location of each target tract as compiled by one observer. Each figure shows in colour the region where 70% or more of the 20 cases had evidence of a white matter tract within a given voxel. The extent of agreement above 70% is indicated by colour gradation such that areas in yellow (subgenual, Fig. 3a), turquoise (retrosplenial, Fig. 3b) and light green (parahippocampal, Fig. 3c) had the highest level of agreement across cases, and those areas in red (subgenual, Fig. 3a), royal blue (retrosplenial, Fig. 3b) and dark green (parahippocampal, Fig. 3c) were closer to the 70% threshold. Note that in the Supplemental Fig. 2, we plot the Dice coefficient (Dice, 1945) of similarity of the probabilistic overlap maps from each experimenter, for a range of thresholds. We note that while the threshold of 70% may appear somewhat arbitrary, with more lenient thresholds, the results would not be very different—since the Dice coefficients are relatively stable between 10 and 70%.

When these tract estimates are overlaid on one another (Fig. 4) it becomes evident that while there is much overlap they also often occupy adjacent space. All three subdivisions involve extensive areas of white matter above the corpus callosum, though the parahippocampal (unrestricted) subdivision does not extend as far rostral as the retrosplenial and subgenual subdivision. In addition, the retrosplenial subdivision is largely located lateral to the subgenual subdivision (Figs. 1, 3 and 4), while the parahippocampal subdivision is slightly more lateral than the retrosplenial subdivision, despite their overlap. These differences are particular evident when comparing the MNI coordinates shown in Fig. 4. Comparisons between the subgenual subdivision and the unrestricted parahippocampal subdivision above the more rostral corpus callosum are also striking as the unrestricted parahippocampal bundle consistently occupied the more lateral position, which was also often slightly more ventral. A consequence was that the subgenual and unrestricted parahippocampal subdivision bundles barely overlapped at these AP levels, i.e., they were side by side, with the subgenual subdivision the more superior (Fig. 3).

Both the retrosplenial and parahippocampal subdivisions have fibres in the temporal lobe that are close to the splenium, but the parahippocampal fibres are again located slightly more lateral to those of the retrosplenial subdivision. The parahippocampal subdivision also extends further forward within the medial temporal lobe than the retrosplenial subdivision. These latter differences are equally evident when using the restricted parahippocampal reconstructions (Supplemental Figs. 3 and 4)

A further way to test for changes across the three derived tracts is to compare their mean fractional anisotropy (FA, Table 1, upper right division) and their mean radial diffusivity (RD, Table 1, lower left division). Separate sets of comparisons were made for each observer (paired *t*-tests, alpha *p*<0.0056). The observers found that in both hemispheres the subgenual subdivision had different FA (*p*<0.0056) and RD (*p*<0.001) properties than the retrosplenial subdivision (Table 1). Likewise, in both hemispheres the mean FA and RD scores for the retrosplenial subdivision were significantly different (*p*<0.001) from those of unrestricted parahippocampal subdivision (Table 1). The same differences between the retrosplenial subdivision and the parahippocampal subdivision were again found for the ‘restricted’ reconstruction (Supplemental Table 1). In contrast, aside from FA in the left hemisphere, none of the FA or RD measures differed between the subgenual subdivision and the unrestricted parahippocampal subdivision (Table 1) at the corrected alpha level. For the restricted parahippocampal subdivision (Supplemental Table 2) none of the comparisons with the subgenual subdivision was significant. There were also no significant FA or RD differences at the corrected alpha level when the same subdivision was compared across hemispheres, e.g., right versus left subgenual subdivision (Table 1).

These tract comparisons included an examination of the correlations between the RD and FA measures for the three putative tracts (Table 2). These analyses help to determine whether either RD or FA co-varied across hemispheres or across tract subdivisions (Table 2) and, hence, whether measurements taken in one part of the tract might be representative for other parts of the same tract. For RD, measures for the same tract region consistently correlated across hemispheres for both observers (Table 2, bottom left division, all *p*<0.0056 except for the left and right retrosplenial subdivision which was not significant at the corrected level, but both observers *p*<0.05). A less consistent pattern was, however, found for FA as while the parahippocampal subdivision (unrestricted) correlated across hemispheres (Table 2), the interhemispheric correlations for the retrosplenial subdivision (left against right) were only significant for one observer (Table 2 upper right division). The subgenual subdivision did not correlate significantly across hemispheres.

Correlations were also examined across different tract subdivisions within the same hemisphere (Table 2). For RD, the pattern of results was very similar for the two observers. The left subgenual subdivision and the left retrosplenial subdivision had RD measures that closely correlated (*p*<0.0056), as did the right (and left) retrosplenial subdivision with the right (and left) unrestricted parahippocampal subdivision (both *p*<0.0056). (The corresponding correlation for the left restricted parahippocampal subdivision was not significant—see Supplemental Table 3) No other subdivision correlations within the same hemisphere were significant. For FA, there were no significant correlations (*p*<0.0056) between different subdivisions in the same hemisphere with the sole exception of the right parahippocampal subdivision and the right retrosplenial subdivision, which was significant for one observer only (Table 2, see also Supplemental Table 3). The finding that the preponderance of FA correlations were not significant suggests changing properties along the extent of the tract.

### 3.3 Comparisons between the two parahippocampal subdivision reconstructions

Reconstructing the cingulum fibres in the medial temporal lobe poses particular problems as the fibres are potentially connected with diverse sites in the occipital, parietal, and frontal lobes, and information is potentially lost if these are all grouped together. Consequently the particular placement of any AND and NOT gates can emphasise different sets of interconnections. Two reconstruction algorithms were, therefore, assessed. By placing a NOT gate above the corpus callosum (Fig. 1), the restricted reconstruction should favour more posterior connections within the occipital and parietal lobes. By removing this NOT gate, additional anterior connections should be revealed.

Reconstructions based on the 70% concordance criterion across the twenty cases (from one observer) show the relative locations of the unrestricted (Figs. 3 and 4) and restricted (Supplemental Figs. 3 and 4) parahippocampal subdivision. Not surprisingly, the two reconstructions overlapped considerably around the level of the splenium, although the restricted subdivision extended just a little more lateral. The rostral limits of the restricted and unrestricted parahippocampal subdivisions within the temporal lobe appear essentially the same.

One goal was to determine if different tract properties might be exposed by removing the more anterior, i.e., frontal fibres from this subdivision. In fact, for both observers the FA and RD measures for both the right and left hemispheres differed significantly between the restricted and unrestricted parahippocampal subdivision (all *p*<0.001). Despite these differences, RD correlated between the restricted and unrestricted parahippocampal subdivision (both hemispheres, *p*<0.01), and FA correlated within the right hemisphere (*p*<0.056).

### 3.4 Generic ROIs in standard space

**Fig. 5:**
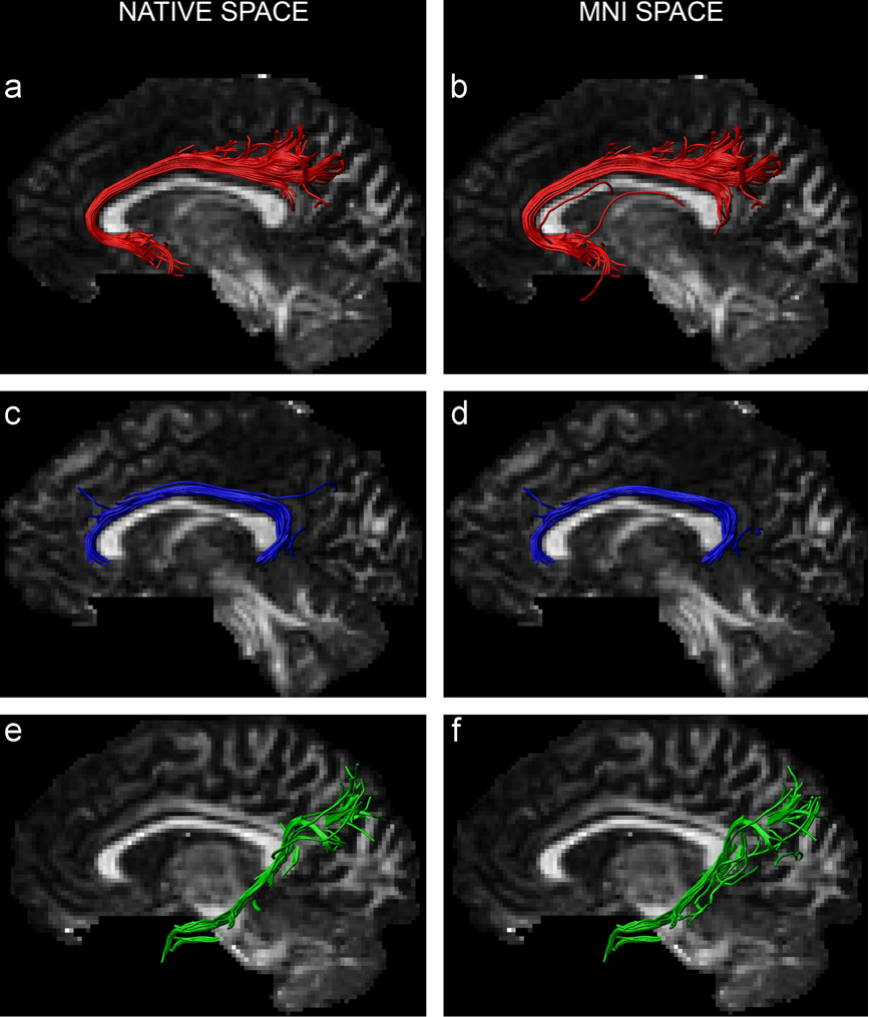
Segmentation of the three subdivisions of the cingulum in a single participant using regions of interest drawn in the participant’s native space (left hand column) and using ROIs that have been nonlinearly warped from MNI space to the participant’s native space (right hand column). (a and b)=subgenual portion; (c and d)=retrosplenial portion; (e and f)=‘restricted’ parahippocampal portion.

The generic ROIs, created in standard space, are available for download at the following link: http://psych.cf.ac.uk/home2/cingulum_rois/, in the NIFTI image format (where 1=ROI voxel, 0=non-ROI voxel). The results obtained from the native-drawn and inverse-warped MNI-drawn ROIs were qualitatively very similar, showing a remarkable degree of homology across methods (see Fig. 5).

## 4 Discussion

The cingulum bundle underlies ‘le grand lobe limbique’ described by Broca (1878), which incorporates the ring of tissue formed by the subgenual, cingulate, and parahippocampal regions. The cingulum itself forms a distinctive white matter tract that appears to almost encircle the corpus callosum and enter the temporal lobe, its belt-like shape giving the tract its name. The present study tested whether the cingulum bundle could be subdivided based on diffusion MRI-based tractography. Three potentially different subdivisions within the cingulum were examined. These subdivisions, which were called the ‘parahippocampal’ ‘retrosplenial’ and ‘subgenual’ subdivisions, were selected because of evidence from axonal transport studies in monkeys indicating that each contains a changing population of white matter (Mufson & Pandya, 1984). In addition, a less constrained reconstruction of the tract was compiled (‘standard cingulum’) to provide a baseline comparison of how the bundle is often portrayed in tractography studies (e.g., Catani et al., 2002; Concha et al., 2005; Gong et al., 2005; Singh & Wong, 2010; Thiebaut de Schotten et al., 2012; Xie et al., 2005). It should be emphasized that previous tract tracing studies with monkeys helped to guide the present investigation, and it was this information that suggested the presence of at least three distinct, but overlapping, subdivisions of the cingulum. The methods did not test whether this represented the optimal number of subdivisions, rather the goal was to determine if there are qualitative differences along the length of the tract.

The present findings strongly suggest that the ‘standard’ tractography cingulum reconstruction is misleading. Instead, it was found that the three main cingulum subdivisions often occupy different space, and that this finding was true even when the subdivisions overlapped, i.e., the tract is topographically arranged so that the white matter from these subdivisions is partially segregated (Figs. 3 and 4). The parahippocampal subdivision is the most lateral, while the subgenual subdivision is the most medial. This separation was particularly evident at those coronal levels where both the subgenual and unrestricted parahippocampal subdivision were present, as there was very little overlap between these two cingulum subdivisions. That the cingulum may have a topographic arrangement has been previously indicated by axonal tracing studies in rhesus monkeys, where fibres from the same sources often aggregate within the cingulum (Schmahmann & Pandya, 2006). For example, thalamic fibres occupy the ventral cingulum bundle, cingulate gyrus fibres occupy the dorsolateral cingulum bundle, and prefrontal fibres occupy the periphery of the bundle (Mufson & Pandya, 1984). While temporal lobe fibres in the monkey brain first traverse the dorsal cingulum, they appear to then aggregate on the medial wall of the tract at more rostral levels (Schmahmann & Pandya, 2006). The present study extended the general topographic principle across species but found differences of detail, e.g., clear evidence that the parahippocampal subdivision remains largely lateral within the bundle (Figs. 1, 3 and 4). These novel findings of how different sets of connections can occupy different locations across the width and depth of the human cingulum open up the possibility of uncovering further dissociations when higher resolution methods are available.

Further support for the conclusion that the tract is not homogeneous comes from the finding that both the subgenual subdivision and the parahippocampal subdivision (unrestricted and restricted) often had mean fractional anisotropy (FA) and radial diffusivity (RD) measures that differed from those of the retrosplenial cingulum. If indeed, the separate subdivision carry connections for different functions, then it is possible that the white matter microstructure is optimized within each subdivision for those particular functions. For example, the axon diameter and myelination (which have competing impacts on diffusion anisotropy (Beaulieu, 2002)), may be differentially optimized according to the pathlength and optimal conduction velocity (Rushton, 1951) needed for the particular task.

Measures of FA and (especially) RD correlated across hemispheres for the same subdivision of the cingulum, but this relationship frequently broke down when correlating either FA and RD scores between different subdivisions within the same hemisphere. This lack of significant correlations across different parts of the standard cingulum is informative as it helps to show that measuring FA (or RD) in one part of the cingulum does not provide a good proxy measure for FA (or RD) in another part of the same tract. These findings, therefore, caution attempts to link white matter measures with aspects of cognition or emotion when the cingulum is reconstructed to give the maximum amount of white matter (e.g., ‘standard’ reconstruction). Differences in subdivision fractional anisotropy could be due to multiple attributes of the tract’s microstructure, and include factors such as packing density, axon diameter, distribution, and myelination (Beaulieu, 2002). These microstructural properties will impact on the capacity of the white matter to transmit electrical impulses. Thus, interpreting changes in FA alone and, in particular, predicting their functional consequences, is challenging (Jones, 2010; Pierpaoli, Jezzard, Basser, Barnett, & Di Chiro, 1996). Advanced microstructural imaging techniques that aim to isolate differences in, for example, axon diameter or myelin content offer promising insights (Alexander et al., 2010; Assaf, Blumenfeld-Katzir, Yovel, & Basser, 2008; Deoni, Rutt, Pierpaoli, & Jones, 2008).

In the reconstructions of the standard cingulum bundle and the three putative subdivisions, it is important to note that common ROIs were used (Fig. 1). Consequently, the subgenual subdivision shared an AND gate with that used to reconstruct the standard cingulum (above the rostral body of the corpus callosum), and the retrosplenial subdivision shared an AND gate with that used for the standard cingulum (above the caudal body of the corpus callosum). Likewise, the parahippocampal subdivision and retrosplenial subdivision reconstructions shared a common AND gate. In fact, the parahippocampal subdivision was reconstructed in two ways (with or without a NOT gate). It might be supposed that this NOT gate would artificially separate the restricted parahippocampal subdivision from the retrosplenial subdivision. In fact, this need not have happened within the temporal lobe as both reconstructions used a common AND gate (just posterior to the splenium) and there was no barrier to stop retrosplenial fibres from occupying the same space as the parahippocampal subdivision (restricted or unrestricted) within the temporal lobe. This use of shared AND gates, therefore, ensured that the pairs of reconstructions could potentially include the same populations of white matter for at least one level. Despite these precautions, the various reconstructed tracts still had different topographies and different FA and RD measures.

The present findings also indicate that only a small proportion of fibres run the apparent length of the tract, otherwise the parahippocampal and subgenual reconstructions would have more closely resembled each other, and both would have closely matched the standard cingulum reconstruction. Similarly, the retrosplenial and parahippocampal subdivision reconstructions would have more closely resembled each other within the medial temporal lobe. This conclusion is noteworthy given that descriptions of the cingulum often imply that many connections traverse much of its length (e.g., Brodal, 1981; Goldman-Rakic et al., 1984; Mufson & Pandya, 1984).

The next step is, therefore, to reconsider the connections in the cingulum bundle and see how they might fit within a revised framework that involves at least three distinct subdivisions within the tract. In reviewing these connections it must be remembered that the only precise information comes from studies of monkey connectivity, and so some cross-species differences are inevitable. It must also be remembered that diffusion MRI methodologies only provide proxy estimates of fibre orientation and cannot determine the direction of a pathway. Given the limited resolution of the technique (2.4 mm isotropic voxels), it is inevitable that voxels contain more than one fibre population (Jeurissen, Leemans, Tournier, Jones, & Sijbers, 2010). Thus, even though leading current methodologies were employed to derive the fibre orientational density function (fODF, Tournier et al., 2004), topological ambiguities remain a challenge for fibre tract reconstruction algorithms, such as differentiating between ‘kissing’, ‘crossing’ and ‘bending’ configurations within an image voxel (see Jones, 2010, Jones & Cercignani, 2010 for reviews of the limitations of the use of diffusion MRI for assessing brain connections in vivo).

Starting with the parahippocampal subdivision, the ‘restricted’ reconstruction helps to highlight inputs to the medial temporal lobe from the posterior cingulate cortex (areas 23, 29, 30, 31), parietal areas such as 7a and LIP (Insausti & Munoz, 2001; Kobayashi & Amaral, 2003, 2007; Morris et al., 1999a; Mufson & Pandya, 1984; Petrides & Pandya, 2006; Suzuki & Amaral, 1994), and from visual areas in the occipital lobe (Suzuki & Amaral, 1994). While, some reciprocal parahippocampal (TH, TF) projections to the posterior cingulate cortices also involve the cingulum (Lavenex, Suzuki, & Amaral, 2002; Yukie & Shibata, 2009), the hippocampal projections from the subiculum to the posterior cingulate region appear to cross directly to the retrosplenial cortex rather than occupy the cingulum bundle (Aggleton, Wright, Vann, & Saunders, 2012). Likewise, although there are direct projections from the hippocampus (CA1 and subiculum) to the frontal cortex (including subgenual and anterior cingulate cortices), these projections rely principally on the fornix and not the cingulum (Aggleton, 2012; Baleydier & Mauguiere, 1980; Barbas & Blatt, 1995; Insuasti & Munoz, 2001; Poletti & Cresswell, 1977; Rosene & Van Hoesen, 1977). Consequently, the large majority of fibres reconstructed above the corpus callosum for the unrestricted parahippocampal subdivision are fibres projecting to the medial temporal lobe. These fibres, which presumably arise from areas 24 and 23, as well as from rostral areas 29 and 30, are located appreciably lateral to the subgenual subdivision. While some projections from the monkey dorsolateral prefrontal cortex and anterior cingulate cortex (e.g., area 24) join the cingulum (Goldman-Rakic et al., 1984; Morris et al., 1999b; Mufson & Pandya, 1984), they principally target the retrosplenial cortex, so that relatively few reach the medial temporal lobe.

In contrast, the ‘retrosplenial cingulum’ contains a great many of the fibres resulting from the reciprocal connections between the prefrontal cortex, anterior cingulate cortex, and posterior cingulate cortex (areas 23, 29, 30, 31) (Goldman-Rakic et al., 1984; Kobayashi & Amaral, 2003, 2007; Mufson & Pandya, 1984; Vogt & Pandya, 1987). The prefrontal connections with the monkey posterior cingulate cortex principally involve the dorsolateral prefrontal cortex, though there are also connections with the orbitofrontal cortex that involve the cingulum (Carmichael & Price, 1995; Goldman-Rakic et al., 1984; Kobayashi & Amaral, 2003, 2007; Petrides & Pandya, 2006; Van Hoesen, Morecraft, & Vogt, 1993; Vogt & Pandya, 1987). A further contribution to the retrosplenial cingulum comes from those projections from the anterior thalamic nuclei and lateral dorsal thalamus to the posterior cingulate cortex that pass around the internal capsule to join the more caudal cingulum above the body of the corpus callosum (Mufson & Pandya, 1984).

The fibres constituting the ‘subgenual cingulum’ have been less well described, though many presumably arise from areas 24, 25, and 32 in the anterior cingulate region (Mufson & Pandya, 1984). Some of these cingulum fibres are thought to reach the insula, uncus, and amygdala (Aggleton, Burton, & Passingham, 1980; Klingler & Gloor, 1960; Mufson & Pandya, 1984). Another group of subgenual cingulum fibres is formed by the cholinergic efferents from the diagonal band and medial septum that go around the genu in the cingulum to innervate cingulate, pericingulate and retrosplenial cortices (Kitt, Mitchell, DeLong, Wainer, & Price, 1987; Seldon, Gitelman, Salamon-Murayama, Parrish, & Mesulam, 1998). The reciprocal connections between the posterior cingulate regions and the anterior thalamic nuclei may constitute another set of subgenual fibres, as in some species a part of this projection passes around the front of the genu (Baleydier & Mauguiere, 1980; Domesick, 1970; Mufson & Pandya, 1984).

There are, therefore, good grounds to see these three subdivisions of the cingulum as containing rather different profiles of connections. This conclusion does not mean that these subdivisions are completely distinct, and examples have repeatedly been given of connections that run through at least two of the subdivisions. Even so, the present findings reveal considerable differences between these three cingulum subdivisions and so have direct neuropsychological implications. It should be added that further qualitatively distinct subdivisions may exist within the cingulum bundle, e.g., within the parahippocampal subdivision.

The status of the cingulum bundle, as measured by diffusion MRI, has been quantified in a growing number of conditions. These conditions include normal aging, Mild Cognitive Impairment, Alzheimer’s disease, schizophrenia, and depression (Cullen et al., 2012; Jones et al., 2005a, 2006; Keedwell et al., 2012; Kubicki et al., 2003; Metzler-Baddeley, Jones, Belaroussi, Aggleton, & O’Sullivan, 2011; Wu et al., 2010; Zhang et al., 2007). It is evident that if the cingulum bundle is not a unitary tract and if it contains distinct populations of white matter at different levels, then the results obtained in any clinical or neuropsychological diffusion MRI study may differ appreciably depending on where and how the tract is reconstructed. Any reconstruction that aims to encompass as much of the tract as possible, i.e., like the ‘standard’ cingulum, is potentially insensitive to change if a disease preferentially targets just one particular set of connections within the tract. Conversely, any reconstruction targeted at just one of the three subdivisions identified in the present study runs the risk of not selecting the most sensitive part of the cingulum bundle. This point is borne out by the failure to find significant correlations for FA across different subdivisions of the cingulum. While some diffusion MRI tractography studies have begun to focus on the status of particular regions within the cingulum bundle, e.g., the subgenual subdivision (Cullen et al., 2010) or the parahippocampal subdivision (Metzler-Baddeley et al., 2011), the present findings provide a strong rationale, along with quantitative guidelines, as to how to extend this practice and so test the specificity of any observed neurological correlations. By subdividing the tract in this way, it should advance our ability to associate neurological changes in the cingulum with the disruption of particular connections and, hence, make more meaningful links with cognition or affect.

## Funding

This work was supported by the Wolfson Trust (JPA, MRMA09R2, to JPA), by the European Commission-funded ‘CONNECT’ project, under the EU-FP7 Future and Emerging Technologies Programme (to DKJ), and by a Wellcome Trust Investigator Award (to DKJ).

## Figures and Tables

**Table 1:** Top right diagonal: Comparisons (paired t tests, two-tailed) between the mean fractional anisotropy (FA) scores of the 20 participants for the three tracts under investigation. A positive t statistic means that the site in the top row has the higher absolute score than the site in the left hand column. The top right diagonal shows the comparisons between all three tracts within the same hemisphere and the comparison for the same tract across the two hemispheres. Bottom left diagonal: Comparisons (paired t tests, two-tailed) between the mean radial diffusivity (RD) scores of the 20 participants for the same three tracts. The bottom left diagonal shows comparisons between all three tracts within the same hemisphere and the comparison for the same tract across hemispheres. Separate results are provided for both observers (KC, RC). Abbreviations: LPH, left parahippocampal cingulum (‘unrestricted’); LRS, left retrosplenial cingulum; LSG, left subgenual cingulum; RPH, right parahippocampal cingulum (‘unrestricted’); RRS, right retrosplenial cingulum; RSG, right subgenual cingulum. The probabilities (^⁎^p≤0.05, ^⁎⁎^p≤0.01, ^⁎⁎⁎^p≤0.001) are indicated, and all results significant at the corrected alpha (p≤0.0056) are in italics.

**Table 2:** Top right diagonal: Correlations (Pearson) between the mean fractional anisotropy (FA) scores of the 20 participants for the three tracts under investigation. Comparisons are shown between all three tracts within the same hemisphere and for the same tract across the two hemispheres. Bottom left diagonal: Correlations (Pearson) between the mean relative diffusivity (RD) scores of the 20 participants for the same three tracts. Comparisons are again shown between all three tracts within the same hemisphere and for the same tract across hemispheres. Separate results are provided for both observers (KC, RC). Abbreviations: LPH, left parahippocampal cingulum (‘unrestricted’); LRS, left retrosplenial cingulum; LSG, left subgenual cingulum; RPH, right parahippocampal cingulum (‘unrestricted’); RRS, right retrosplenial cingulum; RSG, right subgenual cingulum. The probabilities (^⁎^p≤0.05, ^⁎⁎^p≤0.01, ^⁎⁎⁎^p≤0.001) are indicated, and all results significant at the corrected alpha (p≤0.0056) are in italics.

## Footnotes

Appendix A Supplementary data associated with this article can be found in the online version at doi:10.1016/j.neuropsychologia.2012.11.018.
