# Supplementary material for: Distinct subdivisions of the cingulum bundle revealed by diffusion MRI fibre tracking: Implications for neuropsychological investigations
Source: Neuropsychologia. 2013 Jan;51(1):67–78. doi: 10.1016/j.neuropsychologia.2012.11.018 (PMC3611599; doi:10.1016/j.neuropsychologia.2012.11.018)
Supplement: Supplementary file 1 — Supplementary material [file mmc1.doc]

**SUPPLEMENTARY MATERIAL**

| **Sub-Region** | **LSG** | **RSG** | **LRS** | **RRS** | **LPH_U_** | **RPH_U_** |
| --- | --- | --- | --- | --- | --- | --- |
| **Mean DC** | **0.84** | **0.90** | **0.88** | **0.93** | **0.88** | **0.86** |
| **Median DC** | **0.91** | **0.92** | **0.92** | **0.96** | **0.89** | **0.89** |
| **Std(DC)** | **0.16** | **0.08** | **0.11** | **0.07** | **0.07** | **0.11** |

**Supplementary Table 1.**

Dice coefficients for pairwise (observer KC and RC) reconstructions of the cingulum subdivisions.

Abbreviations: DC, Dice coefficient; Std, standard deviation; LSG, left subgenual cingulum; RSG, right subgenual cingulum; LRS, left retrosplenial cingulum; RRS, right retrosplenial cingulum; LPH_U_, left parahippocampal cingulum (‘unrestricted’); RPH_U_, right parahippocampal cingulum (‘unrestricted’).

**Fractional Anisotropy (FA)** Observer 1

Observer 2

|  | **LSG** | **LRS** | **LPHr** | **RSG** | **RRS** | **RPHr** |
| --- | --- | --- | --- | --- | --- | --- |
| **LSG** |  | ***-10.1****** | **1.65** | **-1.89** |  |  |
|  |  | ***-6.92****** | **0.45** | **-1.85** |  |  |
| **LRS** | ***-7.76****** |  | ***9.20****** |  | **2.86**** |  |
|  | ***-5.96****** |  | ***7.45****** |  | **2.08** |  |
| **LPHr** | **1.32** | ***7.61****** |  |  |  | **-1.60** |
|  | **0.27** | ***6.35****** |  |  |  | **-0.97** |
| **RSG** | **-2.79*** |  |  |  | ***-4.92****** | **1.77** |
|  | **-2.73*** |  |  |  | ***-3.57***** | **1.63** |
| **RRS** |  | **-0.39** |  | ***-6.12****** |  | ***7.60****** |
|  |  | **-0.27** |  | ***-4.03****** |  | ***9.55****** |
| **RPHr** |  |  | **-1.87** | **1.84** | ***7.13****** |  |
|  |  |  | **-0.39** | **1.68** | ***6.81****** |  |

**Radial Diffusivity (RD)** Observer 1

Observer 2

**Supplementary Table 2.** Top right diagonal: Comparisons (paired t tests, two-tailed) between the mean fractional anisotropy (FA) scores of the 20 participants for the three tracts under investigation. The results for the parahippocampal subregion (LPHr and RPHr) refer to the ‘restricted’ reconstruction, i.e., when a ‘NOT’ gate is placed above the main corpus callosum (see Figure 1). A positive t statistic means that the site in the top row has the higher absolute score than the site in the left hand column. The top right diagonal shows the comparisons between all three tracts within the same hemisphere and the comparison for the same tract across the two hemispheres. Bottom left diagonal: Comparisons (paired t tests, two-tailed) between the mean radial diffusivity (RD) scores of the 20 participants for the same three tracts. The bottom left diagonal shows comparisons between all three tracts within the same hemisphere and the comparison for the same tract across hemispheres. Separate results are provided for both observers (KC, RC). Abbreviations: LPHr, left parahippocampal cingulum (restricted); LRS, left retrosplenial cingulum; LSG, left subgenual cingulum; RPHr, right parahippocampal cingulum (restricted); RRS, right retrosplenial cingulum; RSG, right subgenual cingulum. The probabilities (*p≤0.05, **p≤0.01, ***p≤0.001) are indicated, and all results significant at the corrected alpha (p≤0.0056) are in italics.

**Fractional Anisotropy (FA)** Observer 1

Observer 2

|  | **LSG** | **LRS** | **LPHr** | **RSG** | **RRS** | **RPHr** |
| --- | --- | --- | --- | --- | --- | --- |
| **LSG** |  | **0.57**** | **0.09** | **0.44** |  |  |
|  |  | **0.47*** | **0.02** | **0.42** |  |  |
| **LRS** | ***0.69***** |  | **0.18** |  | ***0.61***** |  |
|  | ***0.62***** |  | **0.23** |  | **0.49*** |  |
| **LPHr** | **0.20** | **0.35** |  |  |  | **0.34** |
|  | **0.23** | **0.36** |  |  |  | ***0.64***** |
| **RSG** | ***0.67***** |  |  |  | **0.15** | **-0.01** |
|  | ***0.67***** |  |  |  | **0.08** | **-0.04** |
| **RRS** |  | **0.56**** |  | **0.28** |  | **0.37** |
|  |  | **0.54*** |  | **0.29** |  | ***0.65***** |
| **RPHr** |  |  | ***0.71****** | **0.01** | ***0.68****** |  |
|  |  |  | ***0.75****** | **-0.06** | ***0.76****** |  |

**Radial Diffusivity (RD)** Observer 1

Observer 2

**Supplementary Table 3.** Top right diagonal: Correlations (Pearson) between the mean fractional anisotropy (FA) scores of the 20 participants for the three tracts under investigation. Comparisons are shown between all three tracts within the same hemisphere and for the same tract across the two hemispheres. The results for the parahippocampal subregion (LPHr and RPHr) refer to the ‘restricted’ reconstruction, i.e., when a ‘Not’ gate is placed above the main corpus callosum (see Figure 1). A Bottom left diagonal: Correlations (Pearson) between the mean relative diffusivity (RD) scores of the 20 participants for the same three tracts. Comparisons are again shown between all three tracts within the same hemisphere and for the same tract across hemispheres. Separate results are provided for both observers (KC, RC). Abbreviations: LPHr, left parahippocampal cingulum (restricted); LRS, left retrosplenial cingulum; LSG, left subgenual cingulum; RPHr, right parahippocampal cingulum (restricted); RRS, right retrosplenial cingulum; RSG, right subgenual cingulum. The probabilities (*p≤0.05, **p≤0.01, ***p≤0.001) are indicated, and all results significant at the corrected alpha (p≤0.0056) are in italics.

**Supplementary Table 4**

Dice coefficients for pairwise (observer KC and RC) reconstructions of the cingulum subdivisions.

| **Sub-Region** | **LSG** | **RSG** | **LRS** | **RRS** | **LPH_R_** | **RPH_R_** | **LPH_U_** | **RPH_U_** |
| --- | --- | --- | --- | --- | --- | --- | --- | --- |
| **Mean DC** | **0.84** | **0.90** | **0.88** | **0.93** | **0.88** | **0.77** | **0.88** | **0.86** |
| **Median DC** | **0.91** | **0.92** | **0.92** | **0.96** | **0.89** | **0.87** | **0.89** | **0.89** |
| **Std(DC)** | **0.16** | **0.08** | **0.11** | **0.07** | **0.09** | **0.27** | **0.07** | **0.11** |

Abbreviations: DC, Dice coefficient; Std, standard deviation; LSG, left subgenual cingulum; RSG, right subgenual cingulum; LRS, left retrosplenial cingulum; RRS, right retrosplenial cingulum; LPH_U_, left parahippocampal cingulum (‘unrestricted’); RPH_U_, right parahippocampal cingulum (‘unrestricted’); LPH_R_, left parahippocampal cingulum (‘restricted’); RPH_U_, right parahippocampal cingulum (‘restricted’).

**
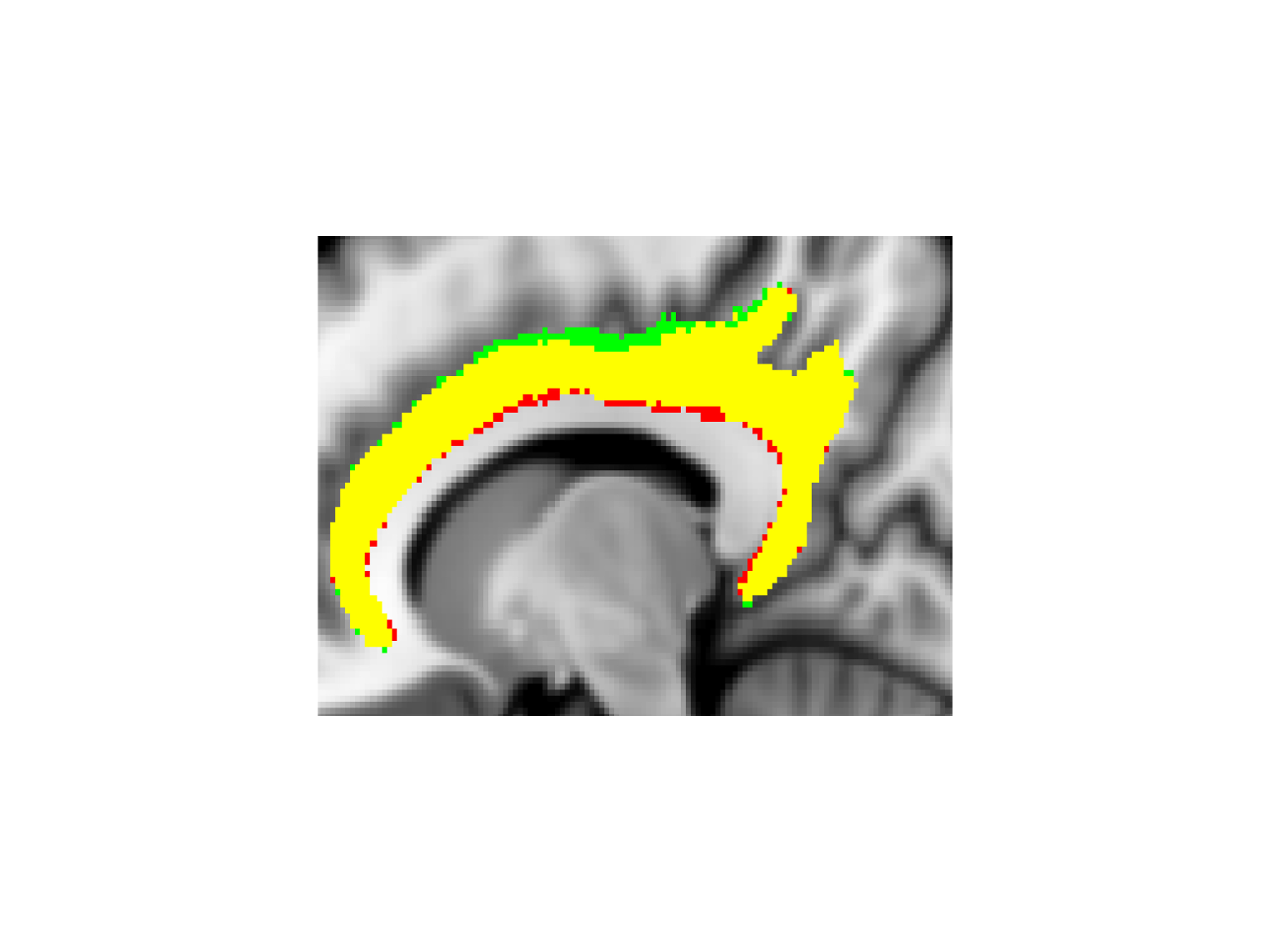
**

**Supplemental Figure 1.**  Probability maps of Observer 1(KC, red) and Observer 2 (RC, green) for the ‘standard cingulum’. The areas in yellow are common to both observers, so that any inter-observer discrepancies are depicted in red and green. The section is in the sagittal plane (rostral cerebrum to the left).


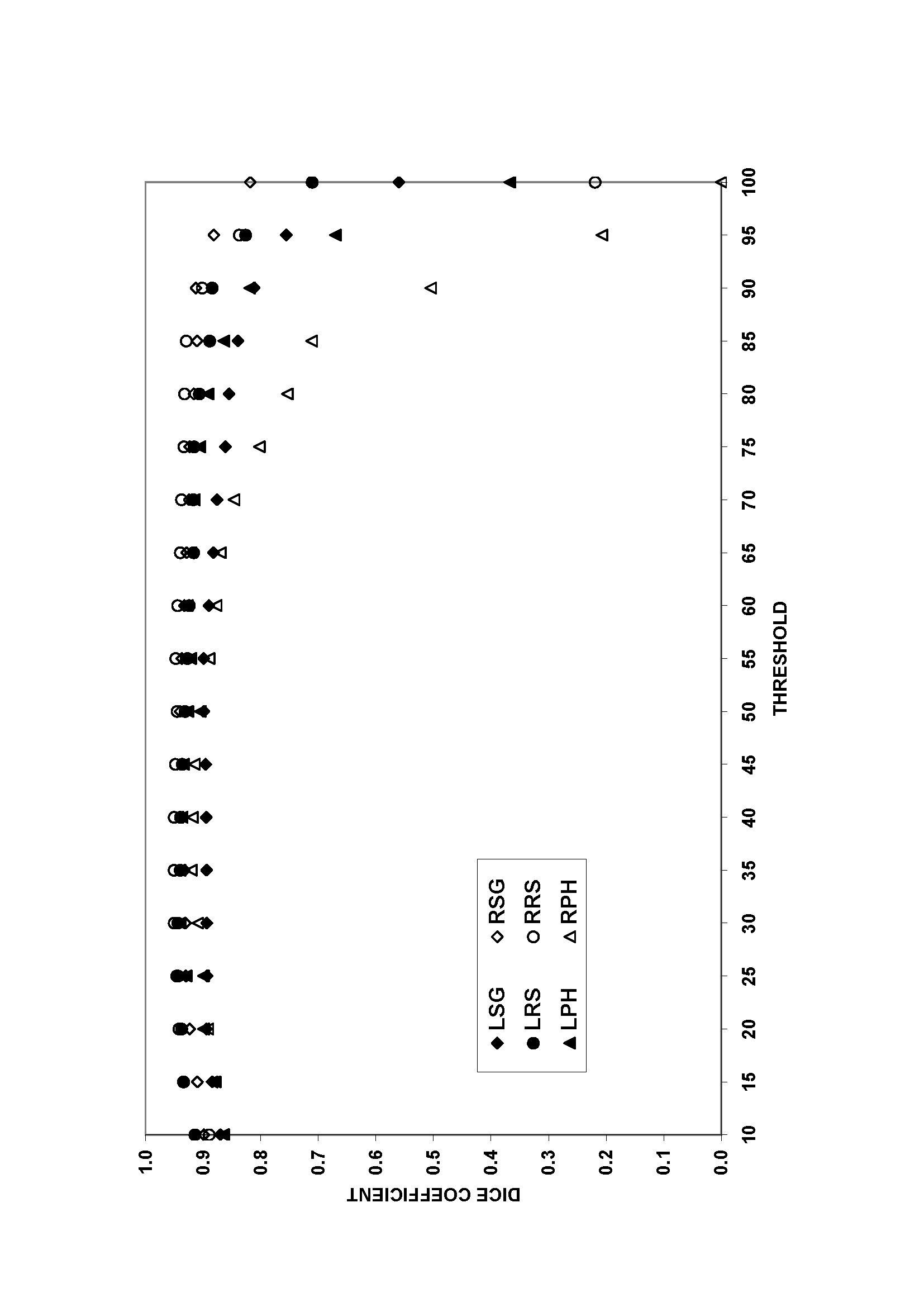


**Supplemental Figure 2:** Inter-experimenter agreement in probabilistic maps of the separate subdivisions of cingulum. For each subdivision, the twenty separate binary maps (one per subject) generated in native space are warped to MNI space and averaged. The plot shows, for each subdivision, the Dice coefficient of inter-experimenter agreement between these averaged maps, as a function of the threshold, (e.g., a threshold of 50% means that a voxel will be non-zero in the probabilistic overlap map if at least 50% of the subjects had at least one streamline intersecting that voxel).

Abbreviations: LSG, left subgenual cingulum; RSG, right subgenual cingulum; LRS, left retrosplenial cingulum; RRS, right retrosplenial cingulum; LPH, left parahippocampal cingulum; RPH, right parahippocampal cingulum.


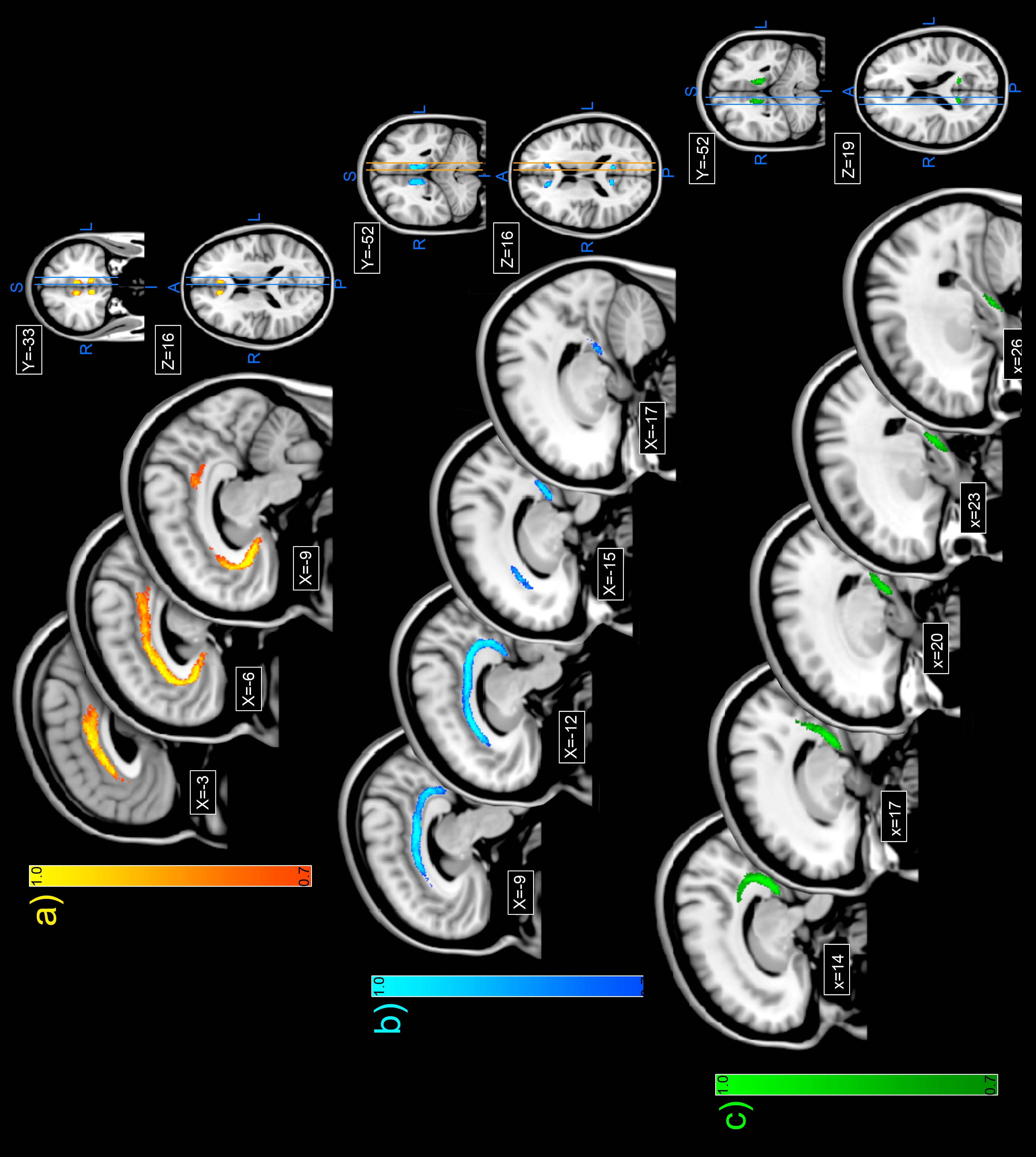


**Supplemental Figure 3.** Population reconstructions for the subgenual cingulum [(a) upper], retrosplenial cingulum [(b)mid], and restricted parahippocampal cingulum [(c)lower]. Each section shows in colour the region where 70% or more of the 20 cases had evidence of cingulum white matter within a given voxel. The extent of agreement above 70% is indicated by colour gradation such that those areas in yellow (subgenual), turquoise (retrosplenial), and light green (restricted parahippocampal) had the highest level of agreement across cases. In contrast, those areas in red (subgenual), blue (retrosplenial), and dark green (restricted parahippocampal) were closest to the 70% threshold. The location of the individual sections is given by their MNI coordinates. The pairs of parallel lines depict the positions of the most medial and most lateral parasagittal sections depicted for that cingulum subregion. All data depicted are from one observer. Abbreviations: A, anterior; I, inferior; L, left; P, posterior; R, right; S, superior.

**
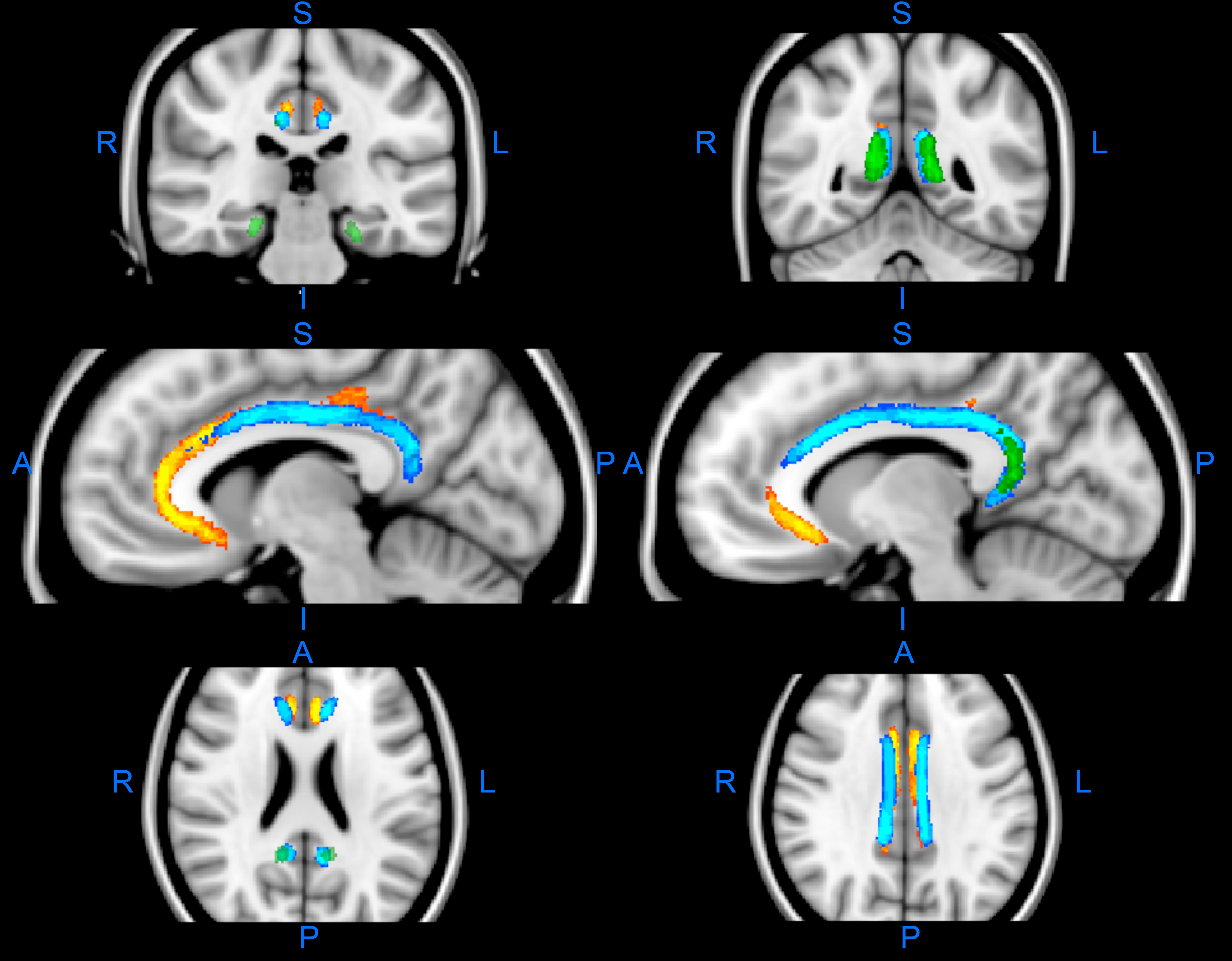
**

**Supplemental Figure 4.** Composite showing the overlay of the subgenual (yellow), retrosplenial (blue), and restricted parahippocampal (green) cingulum reconstructions on each other. All conventions as for Supplemental Figure 3. The sections are in coronal (upper), parasagittal (mid), and horizontal (lower) planes. Abbreviations: A, anterior; I, inferior; L, left; P, posterior; R, right; S, superior.
